# Supplementary material for: Robust structural superlubricity under gigapascal pressures
Source: Nat Commun. 2024 Jul 15;15:5952. doi: 10.1038/s41467-024-49914-6 (PMC11251065; doi:10.1038/s41467-024-49914-6)
Supplement: Supplementary file 1 — Supplementary Information Material [file 41467_2024_49914_MOESM1_ESM.pdf]

Supplementary Information Materials for

Robust Structural Superlubricity under Gigapascal Pressures

Taotao Sun[1,2,3†], Enlai Gao[4†], Xiangzheng Jia[4†], Jinbo Bian[1†], Zhou Wang[1], Ming Ma[1], Quanshui Zheng[1,5,6*], Zhiping Xu[1*]

[1]Center for Nano and Micro Mechanics, Applied Mechanics Laboratory, Department of Engineering Mechanics, Tsinghua University, Beijing, 100084, China.

[2]Railway Engineering Research Institute, China Academy of Railway Sciences Corporation Limited, Beijing, 100081, China.

[3]State Key Laboratory for Track System of High-Speed Railway, China Academy of Railway Sciences Corporation Limited, Beijing, 100081, China.

[4]Department of Engineering Mechanics, School of Civil Engineering, Wuhan University, Wuhan, 430072, Hubei, China.

[5]Center of Double Helix, Institute of Materials Research, Shenzhen International Graduate School, Tsinghua University, Shenzhen, 518055, China.

[6]Institute of Superlubricity Technology, Research Institute of Tsinghua University in Shenzhen, Shenzhen, 518057, China.

*Corresponding author(s). E-mail(s): zhengqs@tsinghua.edu.cn; xuzp@tsinghua.edu.cn;

†These authors contributed equally to this work.

This supplementary information contains Supplementary Notes S1-S7 on

1. Determination of the contact pressure,
2. The pre-cleaning procedure,
3. Friction under high pressures,
4. Breakdown pressures at the tungsten/graphite contact,
5. Characterization of the tungsten tip,
6. Breakdown pressures at defective self-mated graphite contacts,
7. Shear-lag model analysis of graphite tearing,

and Supplementary Figures 1-8, Table 1.

1 Determination of the contact pressure

1.1 Graphite/graphite contact

Contact pressure at the graphite/graphite contact was calculated using the finite element analysis (FEA) (Supplementary Fig. 1(a)). Graphite was modeled as a linear and transversely-isotropic solid. The stress-strain relation is [1, 2]

$$\begin{bmatrix} \sigma_x \\ \sigma_y \\ \sigma_z \\ \tau_{xy} \\ \tau_{xz} \\ \tau_{yz} \end{bmatrix} = \begin{bmatrix} 1060 & 180 & 15 & 0 & 0 & 0 \\ 180 & 1060 & 15 & 0 & 0 & 0 \\ 15 & 15 & 36.5 & 0 & 0 & 0 \\ 0 & 0 & 0 & 440 & 0 & 0 \\ 0 & 0 & 0 & 0 & 4.5 & 0 \\ 0 & 0 & 0 & 0 & 0 & 4.5 \end{bmatrix} \begin{bmatrix} \varepsilon_x \\ \varepsilon_y \\ \varepsilon_z \\ \gamma_{xy} \\ \gamma_{xz} \\ \gamma_{yz} \end{bmatrix} \tag{1}$$

where $\sigma(\tau)$ (in GPa) and $\varepsilon(\gamma)$ are the stress and the strain, respectively. The tip and substrate materials were also modeled as isotropic solids. The Young's moduli (Poisson's ratios) of the tungsten, silica, and silicon are 405 GPa (0.28), 73 GPa (0.17), and 169 GPa (0.28), respectively. Normal and tangential interactions at the mesa/-substrate contact were considered. In the normal direction, a linear cohesive model simplified from the Lennard-Jones potential (Supplementary Fig. 1(a), detailed procedures of calculations can be found in [2]) was introduced in FEA to describe the cohesion from the perspective of molecular simulations. The stiffness of the adhesion measured from the relation between stress and interlayer distances is $K_{\mathrm{nn}} = 11.11$ GPa/nm. The breakdown stress is 0.4 GPa. In the tangential direction, a friction coefficient of µ= 0.001 and a peak shear stress of $\tau_{\mathrm{s}} = 57$ kPa [3] were used to model the shear response. Other contacts are tied in FEA because no relative displacement was identified in the experiments. The geometry was meshed using three-dimensional solid elements (C3D8) and the mesh was refined at the contact area to ensure the convergence of numerical calculations. The model was constructed in consistency with the experimental setup, where the radius of the tungsten tip is 3.5 µm and the thicknesses of the graphite mesa and the mechanically exfoliated graphite substrate are 150 nm and 20 nm, respectively (Supplementary Fig. 2). Supplementary Fig. 1(b) presents a contour of the pressure distribution at the graphite/graphite contact under the load of 10 mN. The critical pressure is valued as the peak stress (9.45 GPa) at the contact. The effective contact radius is 0.8 µm (Supplementary Fig. 1(b)).

1.2 Tungsten/graphite contact

Contact pressure at the tungsten/graphite contact was also calculated using FEA (Supplementary Fig. 1(c-e)). Material parameters remain the same as those introduced in the previous subsection. The cohesive model allows separation and shear at the tip/substrate contact, whereas other contacts are tied according to our experimental observation. The model was meshed into three-dimensional solid elements (C3D8), and the mesh was refined at the contact to ensure the convergence of numerical calculations. The radius of the tungsten tip is 1.75 µm and the thicknesses of the graphite layer and the silica film are 10 nm and 300 nm, respectively. The critical pressure is valued as the peak stress (3.74 GPa, Supplementary Fig. 1(e)) at the contact under

the experimentally determined critical load (0.2 mN). The effective contact radius is 0.16 μm.

A series of sliding tests were carried out experimentally (Supplementary Note 3). To simplify the calculations for these tests, the contact stress was also analyzed using the analytical Hertz model [4], that is

$$\frac{1}{E} = \frac{1-\nu_1^2}{E_1} + \frac{1-\nu_2^2}{E_2} \tag{2}$$

$$a = \left(\frac{3WR}{4E}\right)^{\frac{1}{3}} \tag{3}$$

$$P = \frac{3W}{2\pi a^2} \tag{4}$$

where $E_1 = 405$ GPa ($\nu_1 = 0.28$) and $E_2 = 73$ GPa ($\nu_2 = 0.17$) [2] are the elastic moduli (Poisson's ratios) of the tungsten tip and substrate, respectively. W is the normal load applied to the graphite substrate, R is the radius of the tip, a is the radius of the effective contact region as defined in Eq. 3 (Supplementary Fig. 1(d)), and P is the peak contact pressure in the contact region. Comparison with the FEA results (Supplementary Fig. 1(e)) shows that the prediction of the Hertz model using the parameters of silica (the oxide layer) is more consistent than that using the parameters of silicon.

2 The pre-cleaning procedure

To address the effect of intercalant molecules at the interface, which is unavoidable during sample preparations in ambient conditions, we carried out a pre-cleaning step by sliding the contact in a large area of the substrate before our tests were made in the smaller central region. During the cleaning procedure, friction at the mesa/substrate contact decreases with the sliding cycles and ends in a stable state after about 15 cycles (Supplementary Fig. 3(b)). After the pre-cleaning step, surface roughness (the standard deviation of the height) of the graphite substrate decreased from 1.02 nm to 0.22 nm (Supplementary Fig. 3(c)). The phase contrast image shows a clear difference between the cleaned and the outside regions. The phase of the debris piled at the boundary of the sliding region (Supplementary Fig. 3(a)) is similar to that of the contaminants (Supplementary Fig. 3(a)), which indicates that the debris origins from substrate contaminants.

For the tungsten/graphite experiments (Fig. 2(d)), no obvious phase contrast was characterized between the sliding and outside regions (Supplementary Fig. 3(d)), suggesting a clean substrate. The graphite flakes characterized after sliding tests suggest that the debris comes from the rupture of graphite beyond the breakdown pressure (Supplementary Fig. 3(e)).

3 Friction under high pressures

3.1 Friction of the graphite/graphite contact

The friction of the SSL graphite/graphite interface under high pressures was measured by using a home-built two-dimensional force sensor [5]. The friction force was calculated from the area enclosed in the friction loop divided by the sliding distance (Supplementary Fig. 4. The friction of the self-mated graphite/graphite contact was investigated under a normal load ranging from 0.25 mN to 2.5 mN, which corresponds to pressure from 1.46 GPa to 5.72 GPa, respectively. The loading amplitude is limited by the range of the force sensor. The friction coefficient is measured as $\sim 10^{-5}$, indicating a robust SSL state under gigapascal pressures. Notably, previous studies found that the edge of the mesa/substrate contact contributes the most to the friction force [6], whereas the friction force of the interior region of the contact is nearly zero and remains unchanged under variable pressure, verifying the SSL state of contact.

3.2 Friction of the tungsten/graphite contact

Friction at the non-self-mated tungsten/graphite contact under high pressures was measured using the same method as that for the SSL graphite/graphite contact. The friction coefficient is $\sim 10^{-3}$ under gigapascal-level pressures (Supplementary Fig. 5).

4 Breakdown pressures at the tungsten/graphite contact

6 samples of tungsten/graphite contacts were prepared to determine the breakdown pressure ($P_{\rm cr}$). For each sample, the normal load increases in a sequence of $0.1, 0.3, 0.5, 1, 2, 3, 5, 7, 9$ and 10 mN. Under each load, the tip slid for 10 cycles before wear was characterized. The sliding distance and velocity in all experiments are set to 30 µm and 10 µm/s, respectively. The value of $P_{\rm cr}$ is defined as peak pressure at the contact just before wear is characterized, which varies from 2.73 to 8.40 GPa with an average value of 5.30 GPa (Supplementary Table 1).

Supplementary Table 1: Parameters and breakdown pressures of tungsten/graphite contacts. R is the tip radius. W, P, and a are the load, pressure, and effective contact radius at the contact as wear is identified. $W_{\rm cr}$ and $P_{\rm cr}$ are the load and pressure at the contact before wear is activated, which is defined as the critical load and breakdown pressure, respectively.

#No.	R (µm)	$W_{\rm cr}$ (mN)	$P_{\rm cr}$ (GPa)	W (mN)	P (GPa)	a (µm)
1	4.43	0.5	2.73	1	3.44	0.37
2	1.5	0.1	3.29	0.3	4.74	0.17
3	1.19	0.3	5.53	0.5	6.56	0.19
4	2.27	0.3	3.60	2	6.77	0.38
5	1.19	1	8.26	2	10.41	0.30
6	1.16	1	8.40	3	12.12	0.34

5 Characterization of the tungsten tip

The tungsten tip was characterized by using optical microscopy (OM) before (Supplementary Fig. 6(a)) and after (Supplementary Fig. 6(b)) wear tests. Neither apparent deformation nor material damage was characterized. Electrochemically etched W tip was characterized by high-resolution scanning electron microscopy (SEM, Supplementary Fig. 6(g)) and atomic force microscope (AFM, Supplementary Fig. 6(c, d)), suggesting a very smooth surface at the apex of the tip (Supplementary Fig. 6(c, d)). X-ray photoelectron spectroscopy (XPS) was employed to characterize the chemistry of the tip. The result shows W, WO_3, and WO_2 components within a depth of 10 nm from the surface (Supplementary Fig. 6(f)). The WO_3 surface exposed to the contact with graphite is chosen for first-principles calculations.

6 Breakdown pressures at defective self-mated graphite contacts

The breakdown pressures of defect-free graphite/graphite contacts are higher than the maximum accessible pressure in our experiments. To study the wear characteristics of the graphite/graphite contact, material imperfections were introduced into the graphite substrate by using the argon plasma under a power of 2 W and a time duration of 10 s before a graphite mesa is transferred to it. The argon plasma treatment could add defects to the SSL system without introducing other chemical species [7]. Raman spectrum characterization was conducted to check the implantation of defects, which shows a detectable intensity of the defective graphene peak at 1350 cm^{-1} (Supplementary Fig. 7(a-c)).

The experimental setup of defective graphite/graphite contact follows that of the defect-free graphite/graphite contact. The sliding distance and velocity are 10 µm and 10 µm/s, respectively. The surface morphology of the defective graphite substrate after sliding tests was characterized by atomic force microscopy (AFM) (Supplementary Fig. 7(d)). Rupture of the graphite substrate is observed inside the track under a pressure of 0.4 GPa. Compared to the defect-free graphite/graphite contact, wear is nucleated at a much lower pressure for defective graphite/graphite contact. Multi-site nucleation of wear is characterized inside the sliding track, which evolves into tearing patterns.

7 Shear-lag model analysis of graphite tearing

Interlayer frictional or shear strength of graphite ($\tau_g = 40$ kPa) [8] is much lower than that of a bonded tungsten/graphite contact under high pressure ($\tau_s = 3.65$ GPa). The shear load transferred between the top-most layer and the rest of the graphite can thus be neglected. The critical size of effective contact can be estimated as

$$l_c = \sigma_s t / \tau_s = 11.2 \text{ nm} \tag{5}$$

where $\sigma_s = 120$ GPa and $t = 0.34$ nm are the tensile strength and the interlayer spacing of graphite, respectively. There are two modes of failure as the contact size l is below or above l_c. MD simulations show that as the contact size ($l = 13.30$ nm) is larger than l_c, graphene will be torn by shear-induced tension in the basal planes. However, for $l = 4.06$ nm $< l_c$, the tungsten-carbon bonds break and the interfacial load transfer is insufficient. The contact size in our experiments was estimated in FEA to be a few hundred nanometers, much larger than l_c. Tearing the graphitic layers during tungsten/graphite contact sliding is expected. Our experiment results confirm the increased width of torn tracks and the size of the torn debris as the size of contact increases with the pressure (Supplementary Fig. 7(e)).

Supplementary Figures and Captions

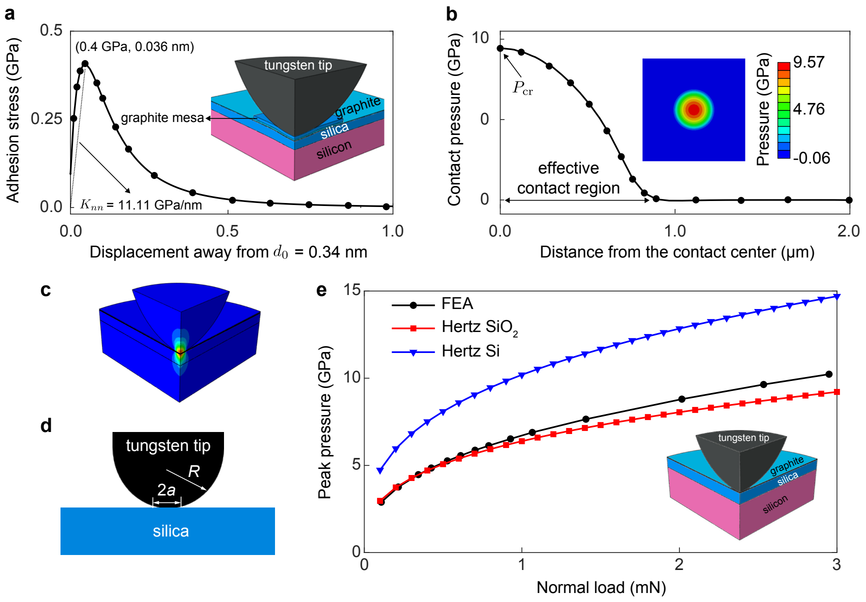

Supplementary Figure 1: Finite element analysis (FEA) of graphite/graphite (a,b) and tungsten/graphite (c-e) contacts. (a) A linear elastic cohesive model to describe mechanical responses of the graphite/graphite contact, and the inset illustrates the FEA model. (b) Pressure distribution at the graphite/graphite contact under a normal load of 10 mN. (c) Stress distribution at the tungsten/graphite contact. (d) The Hertz model. (e) Comparison between FEA and Hertz model predictions. Source data are provided as a Source Data file.

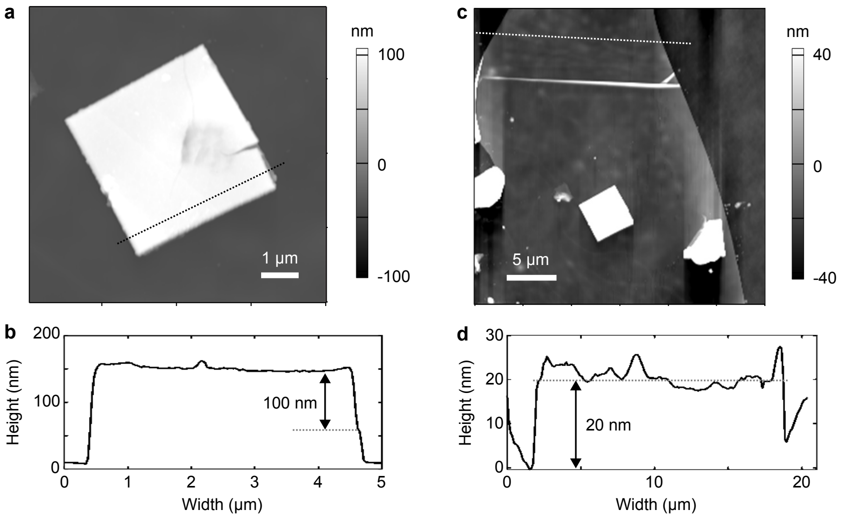

Supplementary Figure 2: Atomic force microscopy (AFM) characterization of graphite thickness. (a, b) Characterization of the graphite mesa. The mesa contains a 100 nm-thick SiO_2 cap and a 50 nm-thick graphite layer. (c, d) Characterization of the graphite substrate. The thickness of the graphite substrate is 20 nm. Source data are provided as a Source Data file.

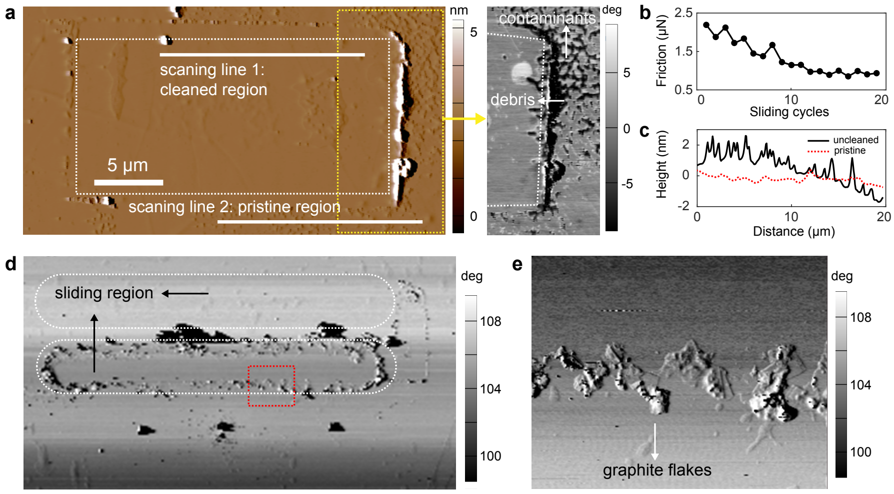

Supplementary Figure 3: Wear tests of the graphite/graphite (a-c) and graphite/-tungsten (d-e) contacts. (a) AFM image of the graphite substrate after a pre-cleaning procedure. The solid lines annotate scanning lines for surface roughness characterization. (b) Friction evolution with the sliding cycles. (c) Height profiles of the graphite substrate at the cleaned central region (pristine) and the outside region (uncleaned). (d) AFM phase image of the graphite substrate after sliding tests of the tungsten/-graphite contact. (e) Magnified view of the debris indicated by the dashed box in (d). Source data are provided as a Source Data file.

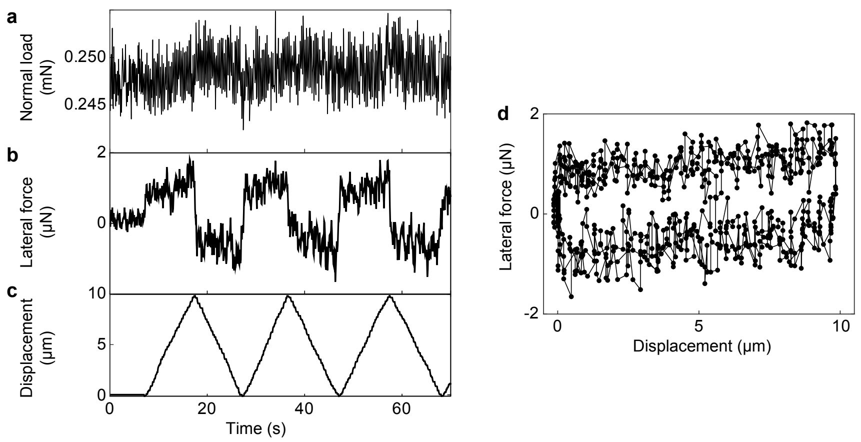

Supplementary Figure 4: Friction measurement at the SSL graphite/graphite contact. (a-c) Normal loads, lateral forces, and displacement measured by a home-built two-dimensional force sensor during the movement of the graphite substrate. (d) Friction loops enclosed by the lateral forces and the sliding distance in forward and backward sliding directions. Source data are provided as a Source Data file.

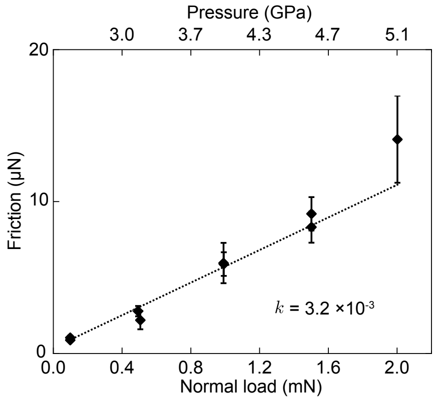

Supplementary Figure 5: Experimental measurement of the friction-load relation of the tungsten/graphite contact. The results of both the loading and unloading processes are summarized. The error bar represents the standard deviation of 10 repeated experiments, and k is the fitting slope. Source data are provided as a Source Data file.

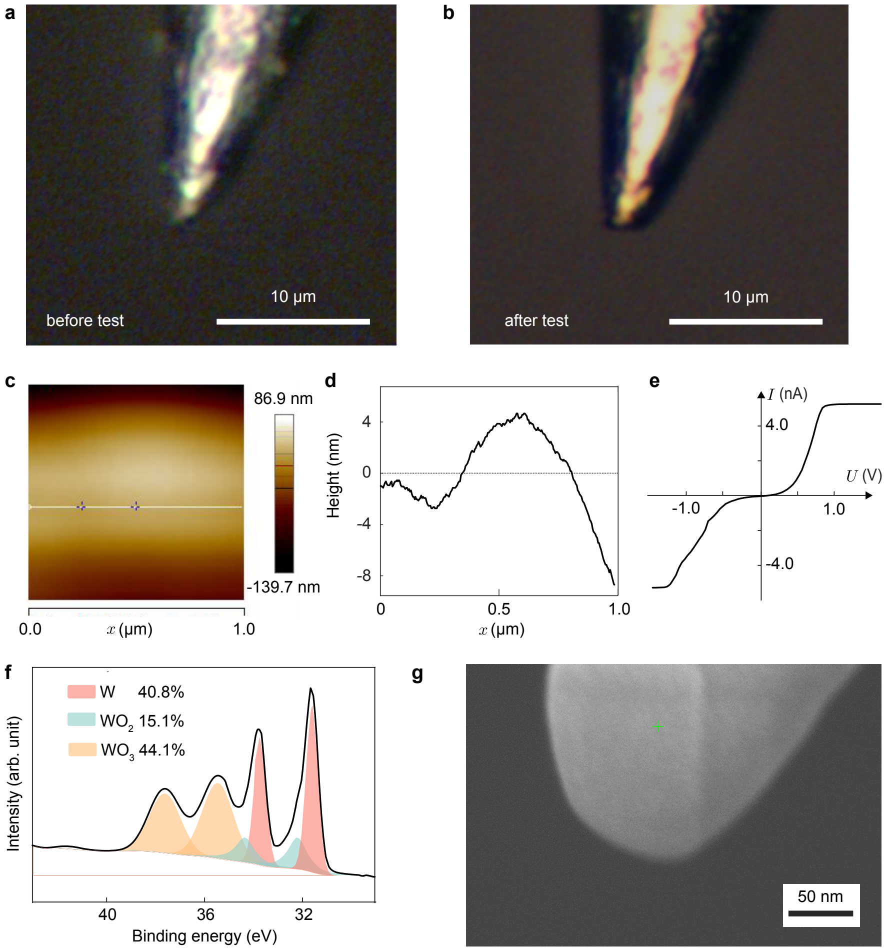

Supplementary Figure 6: Characterization of the tungsten tip. (a, b), Optical microscopy (OM) images of the tip before (a) and after (b) wear tests. (c) AFM height image at the apex of a KOH etched W tip for experiments. (d) A line scan profile taken from the line in (c). (e) I-V curve measured by AFM at the tungsten tip. (f) X-ray photoelectron spectroscopy (XPS) characterization at the tip surface. (g) High-resolution scanning electron microscopy (SEM) image of the electrochemically etched W tip in experiments. Source data are provided as a Source Data file.

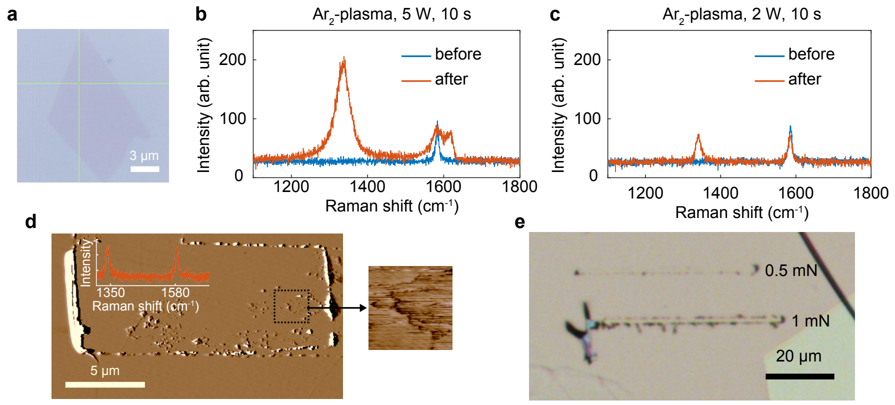

Supplementary Figure 7: Defects in graphite induced by the argon plasma and wear tests at the defective graphite/graphite contact. (a) An OM image of a graphene sheet. (b, c) Raman spectra of samples obtained under argon-plasma treatment of 5 W, 10 s and 2 W, 10 s, respectively. (d) AFM characterization of the morphology of a defective graphite substrate after a sliding test under pressure of 0.4 GPa. Rupture of the graphite substrate is observed inside the track. The inset shows Raman characterization of the plasma-treated graphite substrate before transferring the graphite mesa. (e) Wear of the tungsten/graphite contact under a load higher than the critical value. All sliding tests were conducted at a velocity of 10 μm/s for 10 cycles. Source data are provided as a Source Data file.

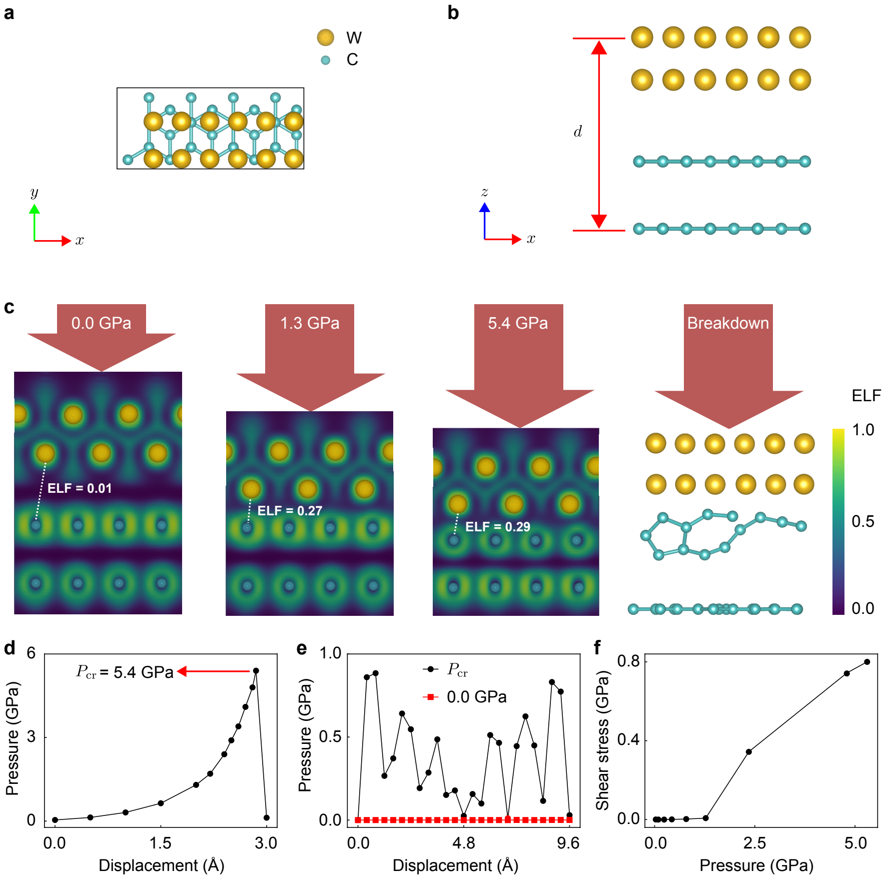

Supplementary Figure 8: Pressure-assisted bonding and tearing-induced wear at the tungsten/graphite interface. (a,b) Model of the tungsten/graphite contact. (c) Snapshots of electron localization function (ELF) at the same slice as the tungsten bulk is forced to move towards graphite, which shows the structural responses and interfacial bonding states evolution under pressure. (d) Pressure-displacement relation obtained from DFT calculations. (e) Shear stress-displacement relation at different pressure levels obtained from DFT calculations. (f) Shear strengths at different normal pressures. Source data are provided as a Source Data file.

References

[1] L. Wang, Q. Zheng, Extreme anisotropy of graphite and single-walled carbon nanotube bundles. Appl. Phys. Lett. **90**(15) (2007)

[2] Z. Liu, J.Z. Liu, Y. Cheng, Z. Li, L. Wang, Q. Zheng, Interlayer binding energy of graphite: A mesoscopic determination from deformation. Phys. Rev. B **85**(20), 205418 (2012)

[3] C.C. Vu, S. Zhang, M. Urbakh, Q. Li, Q.C. He, Q. Zheng, Observation of normal-force-independent superlubricity in mesoscopic graphite contacts. Phys. Rev. B **94**(8), 081405 (2016)

[4] S. Wen, P. Huang, *Principles of Tribology* (John Wiley & Sons, New York, 2012)

[5] T. Sun, Z. Wu, Z. Li, Q. Zheng, L. Lin, A hybrid two-axis force sensor for the mesoscopic structural superlubricity studies. Sensors **19**(15), 3431 (2019)

[6] C. Qu, K. Wang, J. Wang, Y. Gongyang, R.W. Carpick, M. Urbakh, Q. Zheng, Origin of friction in superlubric graphite contacts. Phys. Rev. Lett. **125**(12), 126102 (2020)

[7] A. Eckmann, A. Felten, A. Mishchenko, L. Britnell, R. Krupke, K.S. Novoselov, C. Casiraghi, Probing the nature of defects in graphene by Raman spectroscopy. Nano Lett. **12**(8), 3925–3930 (2012)

[8] G. Wang, Z. Dai, Y. Wang, P. Tan, L. Liu, Z. Xu, Y. Wei, R. Huang, Z. Zhang, Measuring interlayer shear stress in bilayer graphene. Phys. Rev. Lett. **119**(3), 036101 (2017)
